# The genetic and phenotypic variability of interspecific hybrid bermudagrasses (*Cynodon dactylon* (L.) Pers. × *C. transvaalensis* Burtt-Davy) used on golf course putting greens

**DOI:** 10.1007/s00425-016-2573-8

**Published:** 2016-07-22

**Authors:** Eric H. Reasor, James T. Brosnan, Robert N. Trigiano, J. Earl Elsner, Gerald M. Henry, Brian M. Schwartz

**Affiliations:** 1 Department of Plant Sciences, University of Tennessee, 2431 Joe Johnson Dr., 252 Ellington Plant Sciences Bldg., Knoxville, TN 37996 USA; 2 Department of Entomology and Plant Pathology, University of Tennessee, 2505 E.J. Chapman Dr., 370 Plant Biotechnology Bldg., Knoxville, TN 37996 USA; 3 Georgia Seed Development Commission, 2420 S. Milledge Ave., Athens, GA 30605 USA; 4 Department of Crop and Soil Sciences, University of Georgia, 3111 Miller Plant Sciences Bldg., Athens, GA 30602 USA; 5 Department of Crop and Soil Sciences, University of Georgia, 2360 Rainwater Rd., Tifton, GA 31794 USA

**Keywords:** Interspecific hybrid bermudagrass, Off-type grasses, ‘Tifgreen’, ‘Tifdwarf’, Ultradwarf, Putting greens, Genetic instability, Somatic mutation, Aneuploidy

## Abstract

**Some interspecific hybrid bermudagrass cultivars used on golf course putting greens are genetically unstable, which has caused phenotypically different off-type grasses to occur in production nurseries and putting surfaces. Management practices to reduce the occurrence of off-type grasses in putting green surfaces and the effect they can have on putting quality and performance need to be researched until genetically stable cultivars are developed.**

Golf course putting green surfaces in subtropical and tropical climates are typically planted with an interspecific hybrid bermudagrass (*Cynodon dactylon* (L.) Pers. × *C. transvaalensis* Burtt-Davy), because of the superior putting quality and performance of these cultivars. ‘Tifgreen’ was one of the first interspecific hybrids developed for putting green use in lieu of common bermudagrass. However, off-type grasses began appearing in established Tifgreen stands soon after commercial release. Off-type grasses are those with different morphology and performance when compared to the surrounding, desirable cultivar. Off-types have the potential to decrease surface uniformity, which negatively affects putting surface quality. However, several unique off-types from Tifgreen have been selected as commercial cultivars, the first being ‘Tifdwarf’; then ‘Floradwarf’, ‘MS-Supreme’, ‘Pee Dee-102’, and ‘TL-2’, identified later. The cultivars ‘Champion Dwarf’, ‘P-18’, ‘RJT’, and ‘Emerald Dwarf’ were subsequently selected as off-types in Tifdwarf. The naturally occurring off-types and cultivars that have been identified within the Tifgreen family have widely differing phenotypes; however, they are reported to be genetically similar, supporting the hypothesis that their occurrence is a result of somatic mutations. Genetic instability in currently available commercial cultivars is likely to lead to the continued presence of off-types in production nurseries and putting greens. Additional research is needed to understand the nature of genetic instability in Tifgreen-derived cultivars and how to manage its consequences to develop new cultivars, but also strategies for eradication of off-types in pedigree nursery production and end-site putting greens.

## Introduction

Interspecific hybrid bermudagrass (*Cynodon dactylon* (L.) Pers. × *C. transvaalensis* Burtt-Davy) is widely used on turfgrass playing surfaces for sports, particularly golf (Beard 2002). In 2007, bermudagrass was grown on 32 % of the total golf course acreage in the US, and 80 % of putting green acreage in the southern agronomic region (Lyman et al. 2007). The use of sterile, triploid interspecific hybrid bermudagrasses on putting greens began with the development of ‘Tiffine’ (Hein 1953). A later interspecific hybrid, ‘Tifgreen’, improved putting quality, because it could be maintained at lower mowing heights while sustaining optimum leaf density and canopy coverage (Burton 1964; Hein 1961). Shortly, after its commercial release, off-types (grasses with differences in morphology and performance when compared to the surrounding desirable cultivar (Caetano-Anollés 1998; Caetano-Anollés et al. 1997)) began appearing in established putting greens (Burton 1966a; Burton and Elsner 1965).

**Fig. 1:**
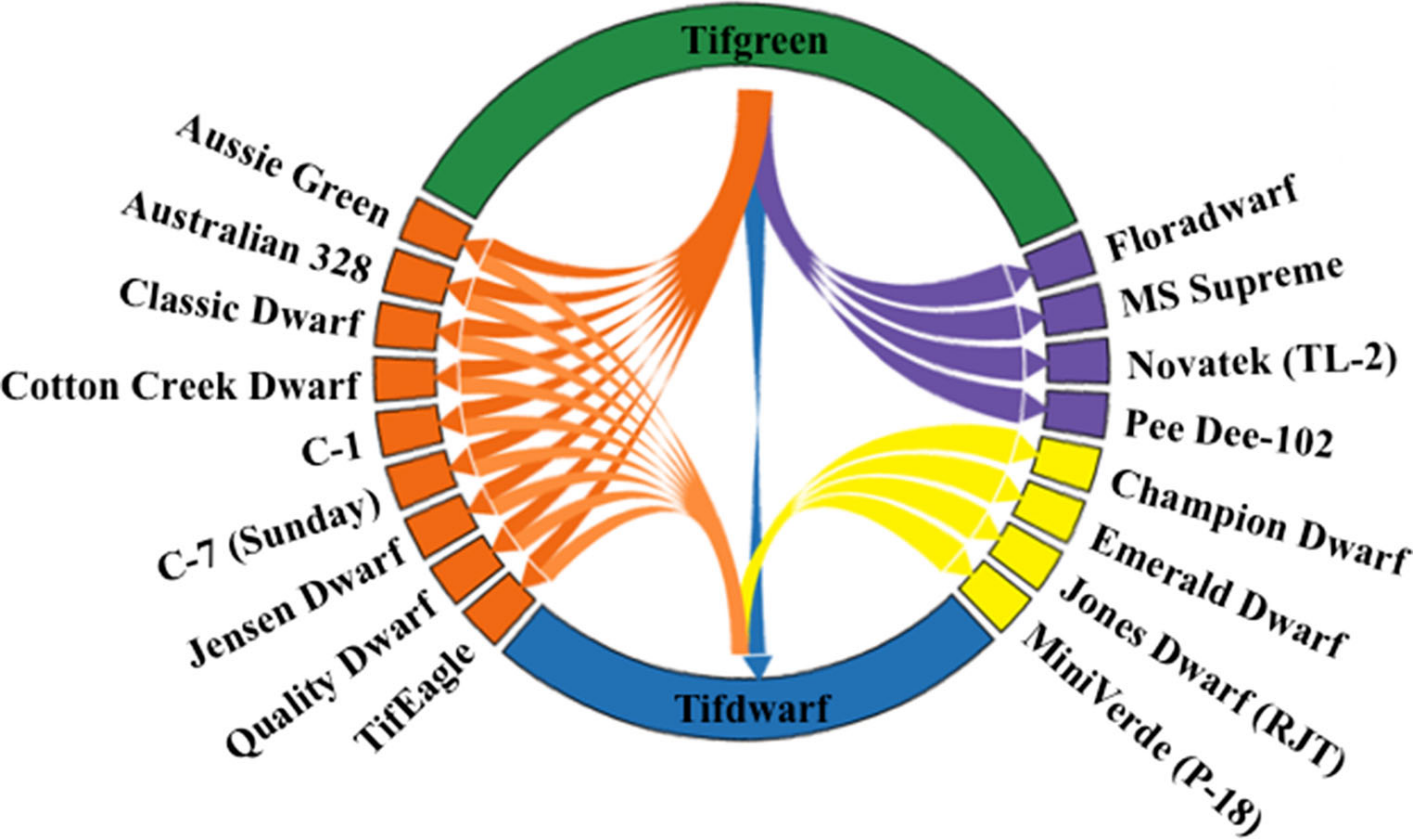
Current understanding of the lineage among accessions of interspecific hybrid bermudagrasses (*Cynodon dactylon* (L.) Pers. × *C. transvaalensis* Burtt-Davy) used on golf course putting greens. The cultivars represented by *blue*, *yellow*, and *purple colors* are those with lineage explicitly reported either in the scientific or in patent literature. The cultivars represented by *orange* are those that the true lineage is unknown or are not explicitly reported by scientific or patent literature

These distinct off-type patches were presumably somatic (vegetative) mutations of Tifgreen, and several were selected and later registered or patented as unique cultivars, including ‘Tifdwarf’ (Burton 1966a), ‘MS-Supreme’ (Krans et al. 1999), ‘Floradwarf’ (Dudeck and Murdoch 1998), ‘Pee Dee-102’ (USDA 1995), and ‘TL-2’ (Loch and Roche 2003b) (Fig. 1). Most of these cultivars were darker in color, had greater canopy density, and were able to withstand lower mowing heights than Tifgreen (Burton 1965, 1966a; Burton and Elsner 1965; Dudeck and Murdoch 1998; Krans et al. 1999). The selection of new commercial cultivars from existing greens continued in the late 1980s through the early 2000s with the discovery of bermudagrasses, such as ‘Champion Dwarf’ (Brown et al. 1997), ‘P-18’ (Kaerwer and Kaerwer 2001), ‘Emerald Dwarf’ (Brown et al. 2009), and ‘RJT’ (Jones et al. 2007) (Fig. 1). Because Tifgreen-derived cultivars are still being widely produced and used (Leslie 2013), the occurrence of off-type grasses is likely to continue in production fields and putting surfaces. Identification and rouging of these off-type grasses are essential to maintain pure stands of the desired cultivar. A thorough review of the development and genetic instability of interspecific hybrid bermudagrasses used on putting greens is needed to better design future research, production, and management programs targeted towards maintaining purity in the field.

## History of bermudagrass development for putting greens

### Early cultivars

Tiffine was one of the first bermudagrass cultivars reported to be more suitable than common bermudagrass (*C. dactylon* (L.) Pers.; 2n = 4x = 36) for use on golf course putting greens (Hein 1953). Tiffine was a sterile, triploid (2n = 3x = 27), interspecific hybrid between a tetraploid *C. dactylon* (L.) Pers. cv. ‘Tiflawn’ and a diploid (2n = 2x = 18) *C. transvaalensis* Burtt-Davy (Forbes and Burton 1963; Hein 1953). Dr. Glenn W. Burton with the US Department of Agriculture–Division of Forage Crops and Diseases (later renamed to Agricultural Research Service) developed Tiffine in 1949 in cooperation with the University of Georgia (UGA) at the Georgia Coastal Plain Experiment Station in Tifton, GA (Forbes and Burton 1963; Hein 1953). Hein (1953) reported that Tiffine was selected based on improved color, texture, and growth habit. The cultivar was released in 1953 (Hein 1953) and was established on putting greens throughout the Southeastern US until the release of Tifgreen in 1956.

Dr. Glenn W. Burton also developed Tifgreen bermudagrass in cooperation with UGA at the Georgia Coastal Plain Experiment Station (Hein 1961). Similar to Tiffine, Tifgreen was a sterile, triploid, interspecific hybrid between a *C. dactylon* selection from a putting green in Charlotte, NC and a *C. transvaalensis* breeding line (Burton 1964; Forbes and Burton 1963; Hein 1961). The cross-pollination program between the two *Cynodon* spp. that yielded Tifgreen was initiated in 1951. The resulting interspecific hybrids were tested until the commercial release of Tifgreen in 1956. The fine texture, density, and rapid growth of Tifgreen made it well suited for golf course putting greens (Burton 1964; Hein 1961). Hein (1961) reported that Tifgreen had greater sod density, weed resistance, fine texture, softness, and color compared to common bermudagrass established from seed. Tifgreen survived winters in Manhattan, KS and Beltsville, MD; however, researchers only recommended Tifgreen for use in southern climates where bermudagrasses were normally grown (Burton 1964; Hein 1961). Tifgreen was reported to be susceptible to sod webworm (*Crambus* spp.) damage and injury from 2,4-dichlorophenoxyacetic acid (2,4-D) herbicide applications (Hein 1961), which could negatively affect overall quality.

Genetic instability of Tifgreen gave rise to off-type grasses of variable phenotypes that appeared soon after establishment (Caetano-Anollés 1998; Caetano-Anollés et al. 1997). In many cases, these off-types exhibited superior characteristics and were later propagated and released as commercial cultivars. The vast majority of bermudagrass cultivars established on putting greens since 1960 are genetically related to Tifgreen; therefore, the development and widespread use of Tifgreen formed the foundation of current bermudagrass cultivars used on putting greens today.

### Tifgreen-derived cultivars

Tifdwarf was the first off-type of Tifgreen to be selected, researched, and released as a commercial cultivar, and has since been used on putting greens throughout subtropical and tropical climates. James Moncrief first identified Tifdwarf as one of two vegetative mutations in mature Tifgreen putting greens in Georgia and South Carolina (Burton 1966a; Burton and Elsner 1965; O’Brien 2012). Burton (1964) reported that the mutation from which Tifdwarf was selected might have been present in the first Tifgreen planting stock before it was distributed for experimentation. Tifdwarf was reported to have the same number of chromosomes as Tifgreen, but its phenotype/genotype allowed it to outperform Tifgreen on golf course putting greens (Burton 1965, 1966a; Burton and Elsner 1965). Tifdwarf has a lower growth habit than Tifgreen, which facilitated mowing at heights of 4.76 mm (Burton 1965, 1966a; Burton and Elsner 1965). Burton (1965) reported that Tifdwarf required less frequent mowing and topdressing than Tifgreen, which resulted in reduced maintenance expenses. In addition, Tifdwarf had softer leaves, fewer seed heads, darker green color, and slightly greater winter hardiness than Tifgreen (Burton 1965, 1966a; Burton and Elsner 1965). The genetic instability of Tifdwarf was similar to Tifgreen (Burton 1965, 1966a; Caetano-Anollés et al. 1997; Caetano-Anollés 1998); therefore, widespread use of Tifdwarf, like Tifgreen, facilitated the selection of off-types that were later released as commercial cultivars.

Pee Dee-102 was selected from a mutation in an early planting of Tifgreen at the Pee Dee Experimental Station (Florence, SC, USA). The South Carolina Agricultural Experiment Station (Clemson, SC, USA) released Pee Dee-102 in 1968, and the South Carolina Foundation Seed Association (Clemson, SC, USA) managed the foundation stock. Pee Dee-102 was reported to have smaller leaves and shorter internodes than Tifgreen, which provided an improved putting surface (USDA 1995).

The Florida Agricultural Experiment Station registered Floradwarf bermudagrass as a commercial cultivar after its release in 1995 (Dudeck and Murdoch 1998). It was selected in 1988 as an off-type plant on golf course located in Hawaii and was thought to be a mutation of Tifgreen. There are contrasting reports regarding the phenotypic characteristics of Floradwarf and Tifdwarf. Dudeck and Murdoch (1998) reported that Floradwarf has greater density than Tifdwarf due to shorter stolons, internode length, and leaf length; however, Roche and Loch (2005) reported that Floradwarf and Tifdwarf have similar internode length, stolon diameter, leaf length, and leaf width. Thatch development occurs relatively fast in Floradwarf putting greens, necessitating timely vertical mowing and topdressing (Dudeck 1995; Dudeck and Murdoch 1998). Dudeck and Murdoch (1998) also state that winter overseeding with perennial ryegrass (*Lolium perenne* L.) in Floradwarf greens is hindered due to high canopy density, but roughstalk bluegrass (*Poa trivialis* L.) can successfully be established. Floradwarf is susceptible to dollar spot (*Sclerotinia homoeocarpa* F.T. Bennett), tropical sod webworms (*Herpetogramma phaeopteralis* Guenée), mole crickets (*Scapteriscus* spp.), and sting nematodes (*Belonolaimus longicaudatus* Steiner) (Dudeck and Murdoch 1998).

MS-Supreme is an improved interspecific hybrid bermudagrass selected in 1991 from a Tifgreen putting green originally planted in 1964 at Gulf Shores Golf Club (Golf Shores, AL, USA) and was released by the Mississippi Agricultural and Forestry Experiment Station in 1997. MS-Supreme was selected for high density, fine texture, prostrate growth habit, and tolerance to low mowing heights. Due to the morphology and growth habit of MS-Supreme, management requires an intensive cultivation program for thatch control (Krans et al. 1999). Krans et al. (1999) reported that internode length and stolon diameter of MS-Supreme were shorter than Tifgreen, but not Tifdwarf. To ensure high-quality sod, the foundation stock of MS-Supreme was maintained by the Mississippi Agricultural and Forestry Experiment Station (Krans et al. 1999). MS-Supreme is also registered in Australia under the Australian Plant Breeders’ Rights Registration application number 2002/305 (Loch and Roche 2003a).

TL-2, also known as ‘Novatek’, was selected as a mutant of Tifgreen in 1996 at Novotel Palm Cove in Cairns, Queensland (Loch and Roche 2003b). Loch and Roche (2003b) identified TL-2 due to its dark green color, finer-texture, and greater density when compared to other selections from Tifgreen tested at that time. Roche and Loch (2005) later reported TL-2 to have similar stolon internode length, leaf length, and leaf width compared to Tifdwarf. Tropical Lawns Pty Ltd tested mutant selections and then released TL-2 in 2003 under the Australian Plant Breeders’ Rights Registration name TL-2 (Loch and Roche 2003b; Roche and Loch 2005).

### Tifdwarf-derived cultivars

Champion Dwarf (also known as ‘Champion’) was selected in 1987 as an off-type present in a Tifdwarf putting green originally established in 1969 in Walker County, TX (Brown et al. 1997). The original selection of Champion Dwarf was propagated in greenhouse pots from a single sprig in Bay City, TX. These plants were used to plant larger trays and then to establish the first Champion Dwarf production field. Champion Dwarf has been described as having slower vertical growth in conjunction with lateral growth similar to other *Cynodon* spp. (Brown et al. 1997). Compared to Tifdwarf, Champion Dwarf has higher shoot density and narrower leaves (Brown et al. 1997).

P-18 (hereafter referred to as ‘MiniVerde’) was a bermudagrass selected based on its fine texture, high canopy density, rapid growth rate, and uniform green color. First identified in 1992, MiniVerde was an off-type obtained from a putative Tifdwarf line grown in a greenhouse owned by H&H Seed Company in Yuma, AZ. MiniVerde was reported to exhibit darker color, higher quality, and greater density, as well as a shorter root structure than Tifdwarf (Kaerwer and Kaerwer 2001).

Champion Dwarf and MiniVerde are considered “ultradwarf” bermudagrasses along with Floradwarf. The term “ultradwarf” was first coined in 1995 by Dr. Philip Busey from the University of Florida to describe bermudagrass putting green cultivars with significantly more diminutive morphology than Tifdwarf (P. Busey, personal communication, 2016). The term ultradwarf is now widely used in the turfgrass industry to label such cultivars.

Emerald Dwarf was a selection made in 1992 from a Tifdwarf putting green established in the 1970s. Emerald Dwarf was reported to produce longer roots and more rhizomes than Tifgreen or Tifdwarf, which resulted in higher quality, color, and coverage during transition periods (Brown et al. 2009).

RJT, also known as ‘Jones Dwarf’, was selected from the regrowth of a sod production field that was previously established to Tifdwarf in 1996 (Jones et al. 2007). The selection was based on fine texture, low nutrient requirements, and reduced thatch production compared to the surrounding Tifdwarf (Jones et al. 2007).

### Other cultivars

‘TifEagle’ was an ultradwarf bermudagrass selected in 1990 for its high quality, fine texture, and ability to tolerate low mowing heights common on golf course putting greens. Following testing as TW-72, TifEagle was released by the USDA-ARS and the UGA Coastal Plain Experimental Station in 1997. TifEagle was one of 48 putative mutants resulting from the irradiation of ‘Tifway II’ with 70 grays (7000 rads) of cobalt-60 gamma radiation (Hanna and Elsner 1999). While TifEagle was reported to be derived from Tifway II (Hanna and Elsner 1999); Harris-Shultz et al. (2010) and Zhang et al. (1999), both suggested that TifEagle may have been derived from Tifgreen (or a Tifgreen related plant) due to the high dissimilarity coefficients reported between TifEagle and Tifway II using amplified fragment length polymorphism (AFLP) methodology. Findings of Capo-chichi et al. (2005) and Chen et al. (2009) further support this assertion in that both research teams reported a high degree of genetic similarity between TifEagle and Tifgreen. TifEagle is a vegetatively propagated cultivar reported to produce higher quality putting surfaces than Tifdwarf when mowed daily at 4 mm or less. When compared to Tifdwarf, TifEagle produced fewer seedheads, had a higher tolerance to tawny mole cricket (*Scapteriscus vicinus*), but produced more thatch (Hanna and Elsner 1999). Hanna and Elsner (1999) reported that TifEagle had shorter and narrower leaves than Tifdwarf and produced more stolons. Since its commercial introduction, TifEagle has been distributed under sublicensing agreements that require inspections of growing locations to limit off-types and to provide incentive for qualified producers to promote the use of TifEagle (Hanna and Elsner 1999).

In addition to the above-described cultivars, other off-types of unknown parentage, presumably related to Tifgreen, have been selected from bermudagrass greens and marketed as cultivars with characteristics superior to Tifgreen and Tifdwarf. ‘C-1’ is an off-type bermudagrass selected in 1987 from what was known as “Cotton Creek Dwarf” at Cotton Creek Golf Course (Gulf Shores, AL, USA) (Chapman 2016). ‘C-7’ (also know as ‘Sunday’) was an ultradwarf cultivar selected in 2007 from a C-1 putting green also at Cotton Creek Golf Course. C-7 was reported to have similar internode length to Tifdwarf, but longer leaves (Chapman 2016). Other bermudagrass selections marketed on a more regional basis include ‘Quality Dwarf’, ‘Jensen Dwarf’, ‘Classic Dwarf’, ‘Australian 328’, and ‘Aussie Green’ (D. Roberts and J. E. Elsner, personal communications, 2015). Many bermudagrass cultivars first identified, as off-types in established swards of Tifgreen and Tifdwarf have been commercialized. These grasses had different morphology, color, and performance when compared to the parent cultivar, in which they were first identified.

## The genetic instability of commercial cultivars leading to off-types

Bermudagrass cultivars, such as Tifdwarf, Floradwarf, MS-Supreme, Champion, and MiniVerde, were selected from established swards of Tifgreen or Tifdwarf (Burton and Elsner 1965; Brown et al. 1997; Dudeck and Murdoch 1998; Krans et al. 1999; Kaerwer and Kaerwer 2001). They were identified as off-types in putting surfaces, because of differences in morphology and performance (Caetano-Anollés 1998; Caetano-Anollés et al. 1997). The presence of off-type grasses spurred research exploring the genetic stability of Tifgreen and Tifgreen-derived cultivars.

DNA amplification fingerprinting (DAF) is a method that uses arbitrary oligonucleotide primers to detect polymorphisms among closely related organisms (Caetano-Anollés and Bassam 1993; Caetano-Anollés et al. 1995). DNA amplification fingerprinting and arbitrary signatures from amplification profiles (ASAP) were used to assess the genetic stability of both Tifgreen and Tifdwarf. Caetano-Anollés (1998) analyzed 11 Tifgreen and eight Tifdwarf authenticated accessions collected from the foundation field and plots maintained by university research programs. According to this study, Tifgreen and Tifdwarf were genetically unstable due to 211 out of 619 DAF polymorphic loci (from 15 mini-hairpin primers) identifying differences in all, but one of the Tifgreen/Tifdwarf accessions (Caetano-Anollés 1998). Compared to a previous study (Caetano-Anollés et al. 1997), differences were not evident between nine different ‘Tifway’ accessions using 273 DAF loci. Based on these findings, Caetano-Anollés (1998) concluded that Tifway was 18 times more genetically stable than Tifgreen and Tifdwarf.

A possible explanation for the high genetic instability and off-type occurrence in Tifgreen and Tifdwarf is aneuploidy. Aneuploidy is an abnormal number of chromosomes not due to a difference in the number of complete sets of chromosomes, which is called euploidy (Duesberg and Rasnick 2000). Tifgreen bermudagrass is a sterile, triploid, interspecific hybrid, but it would be possible for aneuploidy within this cultivar to originate through mitosis and vegetative (asexual) reproduction or during meiosis of the original cross between *Cynodon dactylon* and *C. transvaalensis*.

Vegetative reproduction of Tifgreen and Tifdwarf from stolons and rhizomes provides greater opportunities for point mutations to accumulate at higher rates than grasses that reproduce sexually (Caetano-Anollés 1999; Harris-Shultz et al. 2011). Subsequent cultivars selected from somatic mutations of Tifgreen and Tifdwarf (i.e., MiniVerde and Champion Dwarf) are proposed to possess the same level of genetic instability reported by Caetano-Anollés (1998) in Tifgreen and Tifdwarf. This is theorized, because aneuploidy in interspecific triploid hybrids is not a terminal condition and can be exhibited in subsequent generations (Henry et al. 2005). Duesberg and Rasnick (2000) documented that aneuploidy is a source of genetic instability, because the somatic mutations that affect phenotypic characteristics evolve spontaneously.

Meiotic irregularity has also been postulated to result in some superior phenotypic changes in certain accessions of interspecific hybrid bermudagrasses in the past (Forbes and Burton 1963; Henry et al. 2005). Forbes and Burton (1963) stated that the perennial growth type and vegetative reproduction associated with bermudagrass could reduce meiotic regularity, which could lead to aneuploidy (Henry et al. 2005). In addition, triploid species can produce viable aneuploidies (mostly trisomics) that have severe effects on phenotypic traits (Birchler et al. 2001; Bridges 1922; Henry et al. 2005). Blakeslee (1922) reported that a triploid *Datura* species produced 12 trisomics and each one exhibited a different phenotype. Similar results have also been reported in tomato (*Solanum lycopersicum* L.; Lesley 1928), corn (*Zea mays* L.; McClintock 1929), and tobacco (*Nicotiana tabacum* L.; Clausen and Cameron 1944).

Parental lineage may explain why aneuploidy could be exhibited in Tifgreen and not Tifway. Despite the fact that both cultivars are interspecific triploid hybrids of *C. dactylon* and *C. transvaalensis* (Burton 1966b; Hein 1961), different accessions and breeding lines were used to make the crosses that produced Tifgreen and Tifway. Burton (1966b) reported that the male parent of Tifway was a *C. dactylon* (L.) Pers. selection having 36 chromosomes and the female parent was *C. transvaalensis* Burtt-Davy selection with 18 chromosomes. The species that were the male and female parents of Tifgreen are not specified in the literature.

Lack of information regarding the parental lines used to produce Tifgreen is significant in that there are contrasting reports regarding the base chromosome number of bermudagrass. The majority of research suggests that the base chromosome number is nine (Advulow 1931; Bowden and Senn 1962; Brown 1950; Burton 1947; Clayton and Harlan 1970; Darlington and Wylie 1956; Forbes and Burton 1963; Harlan and de Wet 1969; Rita et al. 2012); however, there have been reports that some bermudagrass accessions may possess several fragmented chromosomes (Burton 1947; Hurcombe 1948). Other findings suggest that bermudagrass has a base chromosome number of ten (Hunter 1943; Hurcombe 1947; Rochecouste 1962; Shibata 1957; Tateoka 1954). Forbes and Burton (1963) surmised that these contrasting accounts were the result of counting fragments as whole chromosomes. In addition, de Silva and Snaydon (1995) suggested that variation in chromosome number may be due to growing environment. Given the contrasting reports of the base chromosome number in bermudagrass and the meiotic irregularity of the *Cynodon* spp., the chromosome fragments observed by Burton (1947) and Hurcombe (1948) may have been whole chromosomes. In this scenario, some triploid bermudagrass interspecific hybrids could be aneuploid and subject to genetic instability.

The repeated use of pesticides and plant growth regulators (PGR) could potentially influence aneuploidy (Karp 1994; Capo-chichi et al. 2005; Gadeva and Dimitrov 2008). Capo-chichi et al. (2005) reported that chronic exposure of Champion Dwarf bermudagrass in greenhouse culture to the dinitroaniline herbicides, pendimethalin, and oryzalin, induced the formation of four off-type grasses. Three of the four off-types were triploid and morphologically similar to Tifgreen; however, one off-type was aneuploid with several morphological traits measuring larger than Tifgreen (Capo-chichi et al. 2005). Capo-chichi et al. (2005) suggested that this off-type may have originated from common bermudagrass; however, this was not confirmed. Gadeva and Dimitrov (2008) reported that exposure of *Crepis capillaris* L. to high concentrations of the fungicide iprodione and insecticide propargite led to a strong presence of lagging chromosomes and anti-microtubule activity, which resulted in aneuploidy. Karp (1994) stated that high concentrations of the synthetic auxin, 2,4-D, increased chromosome instability in tissue culture. Choice and concentration of a particular pesticide or PGR can influence chromosome variations in regenerated plants, which are important, because it can lead to modifications of phenotype (Karp 1994). Research regarding pesticides and PGRs as direct mutagens is inconsistent. Moreover, effects of pesticides on aneuploidy have primarily been observed in tissue culture and use of these specific pesticides in bermudagrass production nurseries and putting greens may be limited.

Aneuploidy can also result from meristem chimeric tissues (Zonneveld and Pollack 2012). Chimeras possess at least two genetically distinct kinds of tissue side-by-side, which is the result of spontaneous mutation accumulations and cell layer rearrangements (Harris-Shultz et al. 2011; Skirvin and Norton 2015; Zonneveld and Pollack 2012). Zonneveld and Pollack (2012) suggested that the vegetative propagation of meristem chimeras could lead to aneuploidy in plants. Marcotrigiano (2000) reported that meristem damage can reveal mutations of inner layer cells that were previously isolated to a single cell layer, a phenomenon that has been documented in *Hosta* cultivars (Zonneveld and Pollack 2012). The researchers stated that aneuploidy in the outermost meristem layer was the major contributor to phenotypic differences among *Hosta* cultivars, and as a result, aneuploidy is a source of genetic and morphological diversity within the genus (Zonneveld and Pollack 2012).

Due to their arrangement of genetically distinct tissues, chimeras can only be successfully propagated by asexual techniques that use preformed buds and avoid adventitious buds (Skirvin and Norton 2015). Harris-Shultz et al. (2011) suggested that Tifdwarf and TifEagle are chimeras. Vegetative production procedures (i.e., sod nurseries) and routine low mowing of Tifgreen or Tifgreen-derived cultivars on putting greens have the potential to cause meristem damage, which could expose putative *de novo* mutations once isolated to a single layer (Harris-Shultz et al. 2011). These practices also have the potential to successfully propagate chimeric tissues. It should be noted that putative *de novo* mutations leading to off-types are likely to be more common in production nurseries than putting greens; therefore, mowing practices associated with putting greens are theoretically only a small factor causing genetic instability and off-type occurrence of Tifgreen or the Tifgreen-derived cultivar family (J. E. Elsner, unpublished observations, 2015).

Aneuploidy in *Luzula luzuloides* has been documented in tissue culture (Madej and Kuta 2001). Madej and Kuta (2001) explained that mitotic abnormalities were the main cause of the aneuploidy observed in *L. luzuloides*, but chromosome fusion and fission were also causes. Although true aneuploidy was not reported, Goldman et al. (2004a, b) observed phenotypic and chromosome number variations among TifEagle plants in tissue culture. Only 14 % of the plants regenerated from a single embryogenic tissue were morphologically similar to TifEagle and only 67 % remained triploid (Goldman et al. 2004a, b). The remaining plants were hexaploid with dark green color, wider leaves, and taller (Goldman et al. 2004a, b). Lu et al. (2006) reported similar findings in follow-up studies regenerating TifEagle in tissue culture. The researchers suggested that genotype explained the observed phenotypic variation, but the increase in ploidy was likely an effect of plants regenerating from a single embryogenic tissue (Goldman et al. 2004a, b). Production nurseries mass-produce vegetative material to establish bermudagrass cultivars on golf courses and then allow plants to regenerate from vegetative propagules remaining in the nursery after harvest (e.g., rhizomes). Unless production nurseries are periodically rotated or re-established, the process of harvesting and regeneration can occur repeatedly over time potentially introducing variation in phenotype and chromosome number of these cultivars (Harris-Shultz et al. 2011).

Aneuploidy has been reported in a wide range of plant species, including bermudagrass. Gould (1966) reported B-chromosomes, or accessory chromosomes, in two out of three *C. dactylon* selections. De Silva and Snaydon (1995) documented that 15 % of plants within a sample population of *C. dactylon* were aneuploid. Arumuganthan et al. (1999) reported that Tifgreen has 0.24 pg/2C more nuclear DNA than Tifway. Greater DNA content would support the assertion that Tifgreen contained an extra chromosome and is, therefore, aneuploid. There is evidence to support the possibility that aneuploidy contributes to the genetic instability observed with bermudagrass cultivars derived from Tifgreen. However, extensive cytogenetic research on Tifgreen-derived bermudagrass cultivars is needed to support this idea. Regardless of the origin, genetic instability within the Tifgreen family has led to the presence of off-type grasses in both production nurseries and putting greens. This has spurred molecular genetics research aimed at exploring the origins and genetic diversity of off-type grasses occurring in Tifgreen-derived putting greens and stolon production nurseries.

## Genetic diversity among bermudagrass cultivars used on putting greens

Molecular genetics research in turfgrass is difficult due to the high ploidy levels and complex genomes associated with turfgrass species (Fei 2008); however, diversity among triploid bermudagrass cultivars has been researched. The genetic variation of Tifgreen and Tifdwarf was compared using DAF with arbitrary octamer primers. Dendrograms were generated from an unweighted pair group cluster analysis using arithmetic means (UPGMA) and phylogenetic analysis using parsimony (PAUP). DNA amplification fingerprinting revealed differences between Tifgreen and Tifdwarf with five polymorphisms present among three primer sequences; however, the UPGMA and PAUP analyses demonstrated that the two cultivars were very closely related (Caetano-Anollés et al. 1995). Farsani et al. (2012) were able to use inter-simple sequence repeat markers and a UPGMA clustering method to place Tifgreen and Tifdwarf into separate subgroups under the same cluster. These studies confirm that Tifgreen and Tifdwarf are genetically similar despite having differences in phenotype.

Amplified fragment length polymorphisms have also been used to examine the genetic diversity among bermudagrass cultivars and selections throughout the southern United States (Capo-chichi et al. 2005; Chen et al. 2009; Zhang et al. 1999). A UPGMA dendrogram created from dissimilarity coefficients clustered Tifgreen, Tifdwarf, TifEagle, Floradwarf, Champion Dwarf, and MS-Supreme together (Capo-chichi et al. 2005). Zhang et al. (1999) reported a relative genetic dissimilarity coefficient range of 0.08–0.33 among Tifgreen, Tifdwarf, TifEagle, and Floradwarf, which grouped these cultivars into the same cluster. Chen et al. (2009) reported similar results with Champion, Tifgreen, Tifdwarf, and TifEagle belonging to the same UPGMA cluster group due to more than 90 % genetic similarity among one another. The results of these three studies using AFLP markers are similar to the results of Caetano-Anollés et al. (1995) and Farsani et al. (2012), suggesting that these bermudagrass cultivars are genetically similar and cannot be fully distinguished from one another.

**Fig. 2:**
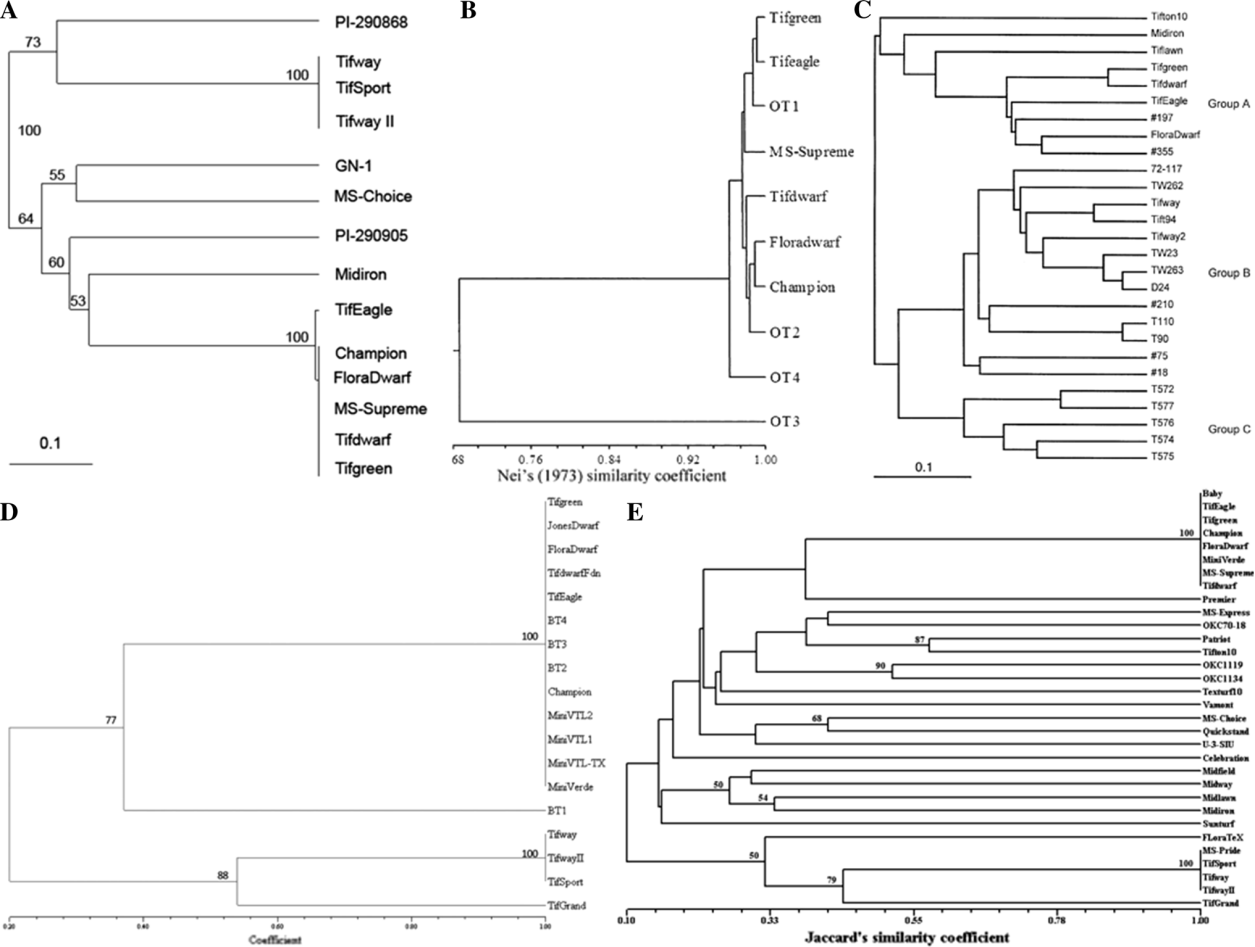
Dendrograms display the genetic relationships among hybrid bermudagrasses (*Cynodon dactylon* (L.) Pers. × *C. transvaalensis* Burtt-Davy) used on golf course putting greens. Dendrograms generated using the UPGMA method from genetic similarity coefficients and SSR, EST-SRR, or AFLP markers. These dendrograms demonstrate that ‘Tifgreen’ and all Tifgreen-derived cultivars cannot be genetically distinguished from one another. **a** Figure reproduced with permission from Crop Science and Kamps et al. (2011). **b** Figure reproduced with permission from Crop Science and Capo-chichi et al. (2005). **c** Figure reproduced with permission from Springer and Zhang et al. (1999). **d** Figure reproduced with permission from the *Journal of American Society of Horticultural Sciences* and Harris-Shultz et al. (2010). **e** Figure reproduced with permission from Crop Science and Wang et al. (2010)

Expressed sequence tags-derived simple sequence repeat (EST-SSR) markers have also been used to examine relationships among Tifgreen, Tifdwarf, TifEagle, Floradwarf, Champion Dwarf, and MiniVerde. Identical alleles were found for the six cultivars, indicating that they were all derived from Tifgreen and could not be differentiated from one another (Harris-Shultz et al. 2010). Wang et al. (2010) reported similar results to Harris-Shultz et al. (2010) using simple sequence repeat (SSR) markers, which grouped Tifgreen, Tifdwarf, TifEagle, Floradwarf, MS-Supreme, Champion Dwarf, and MiniVerde into a single mutation family. The SSR markers used by Wang et al. (2010) identified 22 cultivars derived via the traditional breeding; however, mutation-derived cultivars (such as TifEagle, Floradwarf, MS-Supreme, Champion Dwarf, and MiniVerde) were genetically indistinguishable from each other (Fig. 2). Kamps et al. (2011) also failed to differentiate Tifgreen, Tifdwarf, Champion Dwarf, Floradwarf, or MS-Supreme using SSR markers.

While some previously described SSR markers were not able to identify TifEagle from its relatives, a single amplicon from a primer (Chase 109) has been used to identify TifEagle from Tifgreen- and Tifgreen-derived cultivars (Harris-Shultz et al. 2011; Kamps et al. 2011). Harris-Shultz et al. (2011) reported that the polymorphic fragment amplified by the Chase 109 primer was approximately 142 base pairs larger than the fragment length reported by Kamps et al. (2011). Kamps et al. (2011) suggested that microsatellite instability in plant tissues may be affected by irradiation, similar to mammalian tumors (Haines et al. 2010), potentially explaining why TifEagle is distinguishable from Tifgreen-derived cultivars using the Chase 109 primer. This hypothesis is logical considering that TifEagle has been reported to be a mutant derived from an irradiated Tifway II rhizome (Hanna and Elsner 1999). Simple sequence repeat markers were also reported to identify polymorphic fragments unique to Tifdwarf, TifEagle, and MiniVerde (Harris-Shultz et al. 2011). The SSR markers used to distinguish MiniVerde generated the same polymorphic fragment in shoot and root tissues; however, the markers producing polymorphic fragments specific to TifEagle and Tifdwarf only occurred in shoot tissue. Researchers have also identified a mutating locus of increasing polymorphic fragment length among three Tifdwarf accessions using SSR markers (Harris-Shultz et al. 2011). Certified Tifdwarf collected from Georgia showed one additional allele when compared with Tifgreen, Champion Dwarf, and MiniVerde, which suggested that this mutation may be unique to that location. Champion Dwarf and MiniVerde did not contain the additional Tifdwarf allele; therefore, the mutation producing the additional allele occurred after the mutations that led to the development of those improved cultivars (Harris-Schultz et al. 2011).

Despite having variable morphology and performance, molecular techniques have not clearly distinguished every ultradwarf bermudagrass from one another, or from the cultivars from which they were derived. Figure 2 shows five dendrograms generated from genetic diversity research conducted by Capo-chichi et al. (2005), Harris-Shultz et al. (2010), Kamps et al. (2011), Wang et al. (2010), and Zhang et al. (1999). These dendrograms demonstrate that not all Tifgreen and Tifgreen-derived cultivars can be genetically distinguished from one another, despite variable success SSR markers reported by Harris-Shultz et al. (2011) and Kamps et al. (2011). The ability to identify unique ultradwarf bermudagrass cultivars would facilitate the production of genetically pure planting material, although this purity verification must be performed frequently, because the same pedigree stock production process that led to off-types will be used again. Therefore, if utilized correctly, the ability to identify unique ultradwarf bermudagrass cultivars would improve the uniformity of golf course putting surfaces.

## Genetic analysis of off-types

Phenotype assessments can identify and characterize off-type grasses, but genetic and molecular techniques help explain whether these grasses are mutations or contaminations of registered cultivars (Caetano-Anollés 1998; Caetano-Anollés et al. 1997; Harris-Shultz et al. 2010). Caetano-Anollés (1998) used DAF and ASAP to explore the genetic diversity and origin of 16 off-types present in established Tifgreen and Tifdwarf putting greens on golf courses in the southern US, Hawaii, and Guam. Unweighted pair group cluster analysis and principal coordinate analysis revealed that eight off-types were genetically distinct, but similar to Tifgreen, meaning that they were most likely the result of somatic mutations. The remaining eight off-types yielded genetic distances that were greater than or equal to the differences among the Tifgreen accessions, suggesting that they were the result of sod contamination, which is similar to the previous reports in Tifway (Caetano-Anollés et al. 1997; Caetano-Anollés 1998). The researchers concluded that the presence of off-type grasses in the field was the result of both contaminations as well as somatic mutations (Caetano-Anollés 1998).

Similar to Caetano-Anollés (1998), Harris-Shultz et al. (2010) used EST-SSR makers to identify off-types selected from Tifdwarf and MiniVerde. The EST-SSR markers were successful in identifying whether off-types were genetically similar to Tifgreen (i.e., somatic mutation) or to other cultivars not readily used on golf course putting greens (i.e., contamination) (Harris-Shultz et al. 2010).

Arbitrary primed polymorphic DNA was also used to examine the genetic relationship between Tifdwarf and a single off-type. The amplified products of Tifdwarf and the corresponding off-type sample resulted in a 23 % difference between the two selections, which suggested that these grasses were genetically similar despite having variable morphology (Ho et al. 1997). The amount of genetic similarity reported by Ho et al. (1997), in combination with the results of Caetano-Anollés (1998) and Harris-Shultz et al. (2010), suggests that the off-type studied by Ho et al. (1997) was a somatic mutation of Tifdwarf.

Off-types resulting from somatic mutations of Tifgreen- or any Tifgreen-derived cultivar cannot currently be distinguished from that mutation family by molecular techniques alone; therefore, these off-types cannot be directly linked to parent cultivars, such as Champion Dwarf, MiniVerde, and TifEagle that are mutant selections from within the Tifgreen family as well. New molecular techniques, such as genotyping-by-sequencing (GBS), have the potential to relate off-types to their parent cultivars within the Tifgreen mutation family, because off-types with multiple mutational generations have a decreased certainty of heritage. Information of this nature would further assist in explanation of the origin of off-type grasses in Tifgreen-derived cultivar nurseries and putting surfaces.

## Advances in molecular marker technology for evaluating bermudagrasses

Single nucleotide polymorphisms (SNPs) are mutations that occur between the genomes of related organisms, and are commonly used as molecular markers for genetic research (Fiedler et al. 2015; Mammadov et al. 2012; Vignal et al. 2002; Wang et al. 1998; Yang et al. 2010). Genotyping-by-sequencing described by Elshire et al. (2011) can produce thousands of SNPs, which may be more capable of elucidating differences among bermudagrass cultivars within the Tifgreen mutation family (Elshire et al. 2011; Poland et al. 2012; Poland and Rife 2012). Fiedler et al. (2015) and Poland and Rife (2012) suggested that GBS offers the potential to identify sets of closely linked loci that contribute to phenotypic variation. The ability to connect phenotype to genotype is of great value to researchers to gain a better understanding of the development and progression of bermudagrass cultivars used on golf course putting greens. The connection of phenotype to genotype also has the potential to benefit the development of new cultivars through the conventional breeding techniques.

Elshire et al. (2011) stated that GBS may identify important regions of an organism’s genome that are inaccessible to other molecular marker techniques. For example, Fiedler et al. (2015) used GBS to identify markers in many regions of the switchgrass (*Panicum virgatum*) genome not previously identified by SSR makers. These previously inaccessible areas of a genome are possibly regions of non-coding DNA (Elshire et al. 2011). Elshire et al. (2011) suggested these non-coding, regulatory regions, which control the expression of plant genes responsible for agronomically important phenotypic traits. The ability of GBS to identify these regions of DNA could help researchers to develop molecular markers able to identify genetically similar bermudagrass cultivars and off-type grasses in the Tifgreen family.

The GBS approach is also beneficial, because a reference genome can be developed from only the genomic areas utilized in the procedure (Elshire et al. 2011). This would benefit researchers studying bermudagrass, because a fully sequenced reference genome has not been published. Poland and Rife (2012) suggest that a well-defined reference genome in combination with GBS data makes the development of genetic maps exceptionally straightforward.

## Future insights on the management of off-type grasses

Phenotypic variability of bermudagrass cultivars on putting greens began to be recognized soon after the release of Tifgreen in 1956 and continues to be problematic in ultradwarf greens today. The broad term to describe matrix cultivar variability is “contaminated greens” which includes plants of unrelated off-types from green surrounds, fairways, and production nurseries, as well as off-types related to the matrix cultivar established on the putting surface. Off-types related to the matrix cultivar occur as somatic mutations in both production nurseries and putting greens. When putting surfaces are established with Tifgreen, Tifdwarf, or cultivars with similar morphology, contamination can result from planting stolons infested with matrix cultivar off-types as well as from *de novo* mutations occurring within the putting surface. After several years of putting surface management, these putting surfaces can typically result in significant contamination even if they were initially established with morphologically uniform planting material (J. E. Elsner, unpublished observations, 2015). In contrast, ultradwarf bermudagrass greens have the potential to maintain morphological uniform for many years even though production nurseries have similar mutation frequencies as Tifgreen and Tifdwarf nurseries (J. E. Elsner, unpublished observations, 2015). It has been estimated that the frequency of somatic mutations in ultradwarf production nurseries exceeds three phenotypically different off-types per hectare per year (Harris-Shultz et al. 2010, Caetano-Anollés 1998; Ho et al. 1997; J. E. Elsner, unpublished observations, 2015). Maintaining genetic purity in a production nursery is challenging, because field conditions that allow for profitable production often contrast with management practices that facilitate the identification of off-types through regular inspection. Variation in mowing height, fertility, and irrigation are management tools used to enhance off-type identification.

Off-types must be eradicated from the desirable cultivar before they can expand and be spread across the nursery through cultivation or harvesting procedures. The difficulty in rouging and eradicating off-types in nursery production is likely due to the phenotypic similarities between off-types and commercial cultivars under commonly used nursery management practices. In the event that off-types escape detection and are widely spread during the establishment of new golf greens, the perceived rate and impact of mutation is much higher than on greens planted with morphologically uniform sprigs and which can slowly accumulate somatic mutants over years and decades (J. E. Elsner, unpublished observation, 2015).

Several cultivars are now currently off patent, and the proprietary protection offered by a US Plant Patent is no longer present. These off patent cultivars have the potential to move into the public domain, presenting more difficulties with respect to keeping pedigree stock material off-type free. Use of a cultivar at more production sites makes off-type rouging more difficult. In addition, lack of patent protection may reduce the sale price and profit potential; therefore, reducing economic incentive to remove off-types from planting stock.

Some off-type bermudagrasses within Tifgreen putting surfaces (O’Brien 2012) have exhibited larger internode and leaf lengths, as well as higher canopy height and greater turfgrass cover than commercially available bermudagrass cultivars used on putting surfaces (unpublished data). Off-types with more aggressive, upright growth than commercial cultivars can negatively affect functional and aesthetic putting green quality. Anecdotal observations suggest management practices, such as mowing frequency and height, fertilization, and chemical applications, may be optimized to reduce negative effects of competitive off-types on putting quality. However, research is needed to define agronomic and off-type management strategies and their economic feasibility for golf course putting greens to reduce the negative effects of off-types created from planting contaminated stolons.

Bermudagrass putting greens cover approximately 3642 hectares across the US (Lyman et al. 2007) with 70–80 conversions to ultradwarf bermudagrass occurring each year (Leslie 2013). Tifgreen-derived cultivars are the mainstay of the warm-season golf course putting green market. They are planted worldwide in subtropical and tropical; however, genetic instability can result it phenotypically different off-type grasses in putting surfaces that present significant challenges for golf course superintendents. Interdisciplinary research will be needed to better understand the genetic diversity and instability of bermudagrasses used on putting greens, management strategies to reduce the deleterious effects that off-types pose on putting surface quality, and their economic feasibility of management practices as compared with putting surface replacement.

### *Author contribution statement*

All authors shared responsibility in preparing the manuscript based on their specific areas of expertise.
